# Unusual Manifestation of Live *Staphylococcus saprophyticus, Corynebacterium urinapleomorphum*, and *Helicobacter pylori* in the Gallbladder with Cholecystitis

**DOI:** 10.3390/ijms19071826

**Published:** 2018-06-21

**Authors:** Steffen Backert, Nicole Tegtmeyer, Omar A. Oyarzabal, Dana Osman, Manfred Rohde, Robert Grützmann, Michael Vieth

**Affiliations:** 1 Division of Microbiology, Department of Biology, Friedrich Alexander Universität Erlangen/Nuremberg, Staudtstraße 5, D-91058 Erlangen, Germany; nicole.tegtmeyer@fau.de; 2 Department of Nutrition and Food Sciences, University of Vermont Extension, South Burlington, VT 05405, USA; oaoyarzabal@gmail.com; 3 Institute for Pathology, Klinikum Bayreuth, 95445 Bayreuth, Germany; Dana.osman@outlook.com (D.O.); vieth.lkpathol@uni-bayreuth.de (M.V.); 4 Central Facility for Microscopy, Helmholtz Centre for Infection Research, 38124 Braunschweig, Germany; Manfred.Rohde@helmholtz-hzi.de; 5 Department of Surgery, University Hospital Erlangen, Krankenhausstraße 12, D-91054 Erlangen, Germany; Robert.Gruetzmann@uk-erlangen.de

**Keywords:** gallbladder, cholecystitis, immunohistochemistry, electron microscopy, *Helicobacter pylori*, *Staphylococcus saprophyticus*, *Corynebacterium urinapleomorphum*, protein profiling, 16S rRNA gene sequencing, urease test, CagA, EPIYA, GGT, HtrA

## Abstract

Culture-independent studies have identified DNA of bacterial pathogens in the gallbladder under pathological conditions, yet reports on the isolation of corresponding live bacteria are rare. Thus, it is unclear which pathogens, or pathogen communities, can colonize the gallbladder and cause disease. Using light microscopy, scanning electron microscopy, culture techniques, phylogenetic analysis, urease assays and Western blotting, we investigated the presence of live bacterial communities in the gallbladder of a cholecystitis patient after cholecystectomy. 16S rRNA gene sequencing of isolated bacterial colonies revealed the presence of pathogens most closely resembling *Corynebacterium urinapleomorphum* nov. sp., *Staphylococcus saprophyticus* and *Helicobacter pylori*. The latter colonies were confirmed as *H. pylori* by immunohistochemistry and biochemical methods. *H. pylori* cultured from the gallbladder exhibited both the same DNA fingerprinting and Western *cagA* gene sequence with ABC-type EPIYA (Glu-Pro-Ile-Tyr-Ala) phosphorylation motifs as isolates recovered from the gastric mucus of the same patient, suggesting that gastric *H. pylori* can also colonize other organs in the human body. Taken together, here we report, for the first time, the identification and characterization of a community consisting of live *S. saprophyticus*; *C. urinapleomorphum*, and *H. pylori* in the gallbladder of a patient with acute cholecystitis. Their potential infection routes and roles in pathogenesis are discussed.

## 1. Introduction

Acute cholecystitis is considered a serious, potentially life-threatening complication and is one of the most common surgically treated diseases. Bacterial infection is commonly reported in 50 to 90% of the cases [1,2,3]. Gallbladder infection appears to be typically linked with severe pain, often associated with colics, that can last for several hours. Diverse bacterial flora in the bile and gallbladder of patients with cholecystitis have been described, as detected by PCR (polymerase chain reaction) and culture-dependent methods [1,3,4,5]. The most frequently identified pathogens in biliary infections are Gram-negatives, primarily *Escherichia coli*, *Salmonella enteritidis*, *Acinetobacter baumannii*, *Citrobacter freundii*, *Enterobacter cloacae*, and *Klebsiella* species. Within Gram-positives, *Clostridium perfringens* is most commonly observed. Additionally, in some Asian countries, the presence of *Helicobacter pylori* has been detected infrequently in the gallbladder by PCR [2,6,7,8]. This pathogen is as a type-I carcinogen responsible for gastritis, gastro-duodenal ulcers, and gastric malignancies in the human stomach [9]. However, there are no reports of gallbladder colonization by this pathogen in individuals from Western countries, nor is it understood how potential *H. pylori* infections could lead to gallbladder disease. In this report, an acute case of cholecystitis was presented to our hospital, and after surgical removal, the gallbladder was used for microbial analysis. Here, we present the results of studies using light microscopy, scanning electron microscopy, culture techniques, phylogenetic analysis, urease assays, and Western blotting to investigate the presence of live bacterial communities in the gallbladder of this patient.

## 2. Results

### 2.1. Case Presentation

A 50-year-old male patient with a history of nightly lower abdominal pain for three months was transferred to the emergency room at Erlangen University Hospital. First routine examinations revealed that heart, spine, liver and kidney showed no pathological changes. The patient had no diarrhea, no fever and no apparent changes in blood values. Gastroscopy revealed a moderately chronic slightly active *H. pylori* gastritis as determined by Warthin silver staining and culturing. After collecting a sample by endoscopy, a conventional triple antibiotics therapy for 7 days was prescribed to eradicate gastric *H. pylori*. As the nightly colics continued for the next three weeks, ultrasound diagnostics was performed revealing a thickened gallbladder wall and signs of inflammation, suspicious for cholecystitis. Two gallstones measuring up to 1.5 cm were detected before removal of the gallbladder by standard laparoscopic surgery. Histopathology suggested a microbial infection as the etiology for the observed pathological changes of the gallbladder.

### 2.2. Identification of Live Bacteria in the Gallbladder

**Figure 1:**
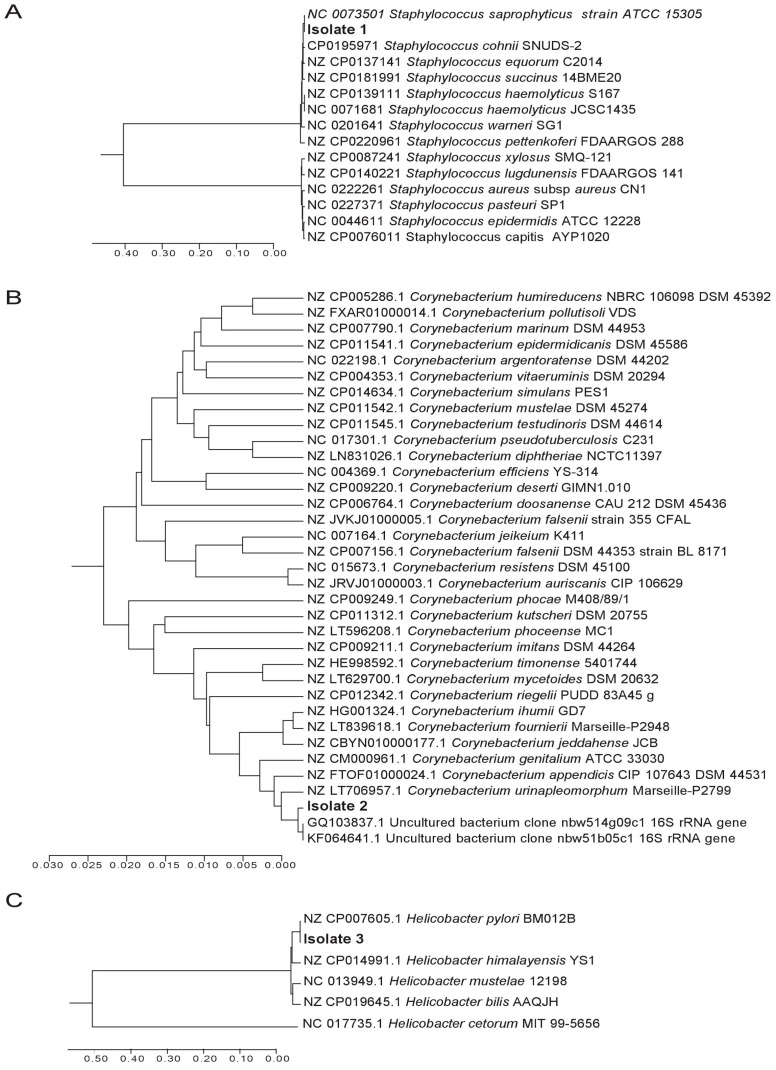
Three isolates of live bacterial species isolated from the gallbladder of a patient with cholecystitis were subjected to 16S rRNA gene sequencing and phylogenetic analysis. The DNA relatedness of sequences with known taxa is shown using the unweighted pair group method and arithmetic average method (UPGMA). The optimal tree with the sum of branch length was 1.04, 0.51, and 1.22 for panels (**A**–**C**), respectively. All positions containing gaps and missing data were eliminated, and the analyses were conducted in MEGA7.

To investigate if viable bacteria were present in the tissue, samples were incubated on different culture plates at 37 °C under aerobic, anaerobic and microaerobic conditions. The most prominent bacterial growth was seen after 3–6 days of incubation on Columbia agar plates supplemented with sheep blood, in a microaerobic atmosphere generated by Campygen^TM^ (Oxoid, Wesel/Germany) [10,11]. Three different colony morphologies were observed, suggesting the presence of at least three different species. Bacterial DNA was isolated from all colonies and subjected to PCR for the amplification of a ~1 kb segment of the 16S rRNA gene using universal primers [12,13]*.* PCR products with the expected sizes were produced from all colonies and were subsequently sequenced. Three different sequences corresponding to the three different colony morphologies were further analyzed by a BLAST (Basic Local Alignment Search Tool) query to determine the identity of the isolated bacteria. Query and reference sequences were aligned using CLUSTAL in MEGA7 (Molecular Evolutionary Genetics Analysis 7) [14] and clustered using the unweighted pair group method and arithmetic average (UPGMA) [15]. Their evolutionary distances were computed using the Maximum Composite Likelihood method, given in the units of the number of base substitutions per site [16]. This analysis confirmed the presence of three different bacterial species. The sequence of Isolate 1 was identical to *Staphylococcus saprophyticus* ATCC 15305 (Figure 1A). Isolate 2 had the closest similarity to uncultured bacterial DNA sequences and resembled *Corynebacterium urinapleomorphum* nov. sp. strain P2799, and this may represent a novel *Corynebacterium* species (Figure 1B), but a more detailed characterization would be needed to confirm this assumption. The 16S amplicon of Isolate 3 was identical to *Helicobacter pylori* strain BM012B (Figure 1C). No other bacterial species were grown from the gallbladder samples. Taken together, the genetic analyses based on the 16S rRNA gene fragments identified the presence of *S. saprophyticus*, *C. urinapleomorphum*, and *H. pylori*.

### 2.3. Microscopic Examination of Gallbladder Bacteria

**Figure 2:**
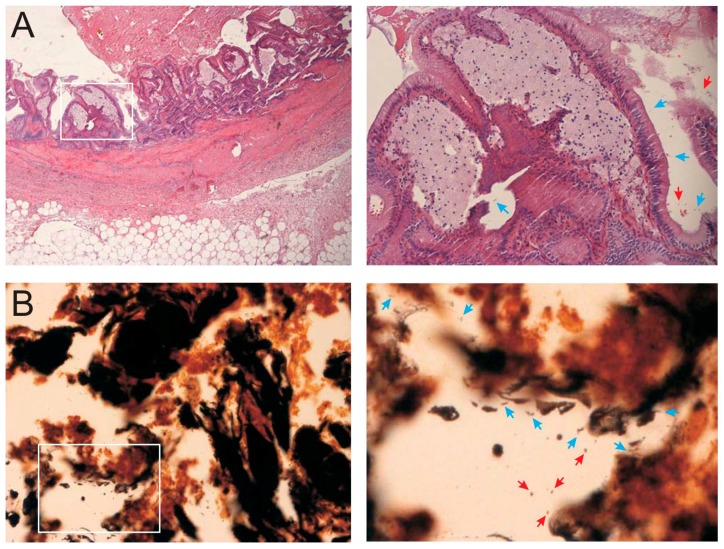

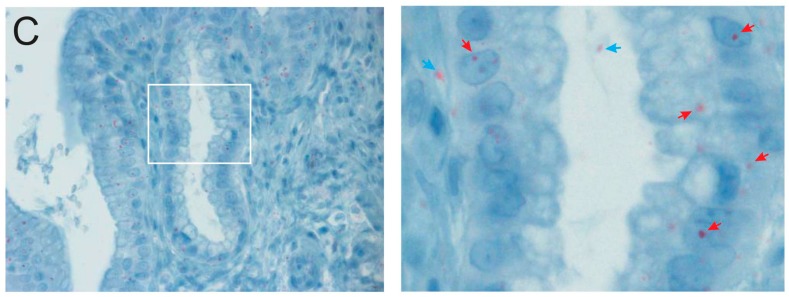
Histological sections from the gallbladder of the patient. The magnification is 40× for the left panels, and a 200× magnification of the identified section (white box) is shown to the right. (**A**) Identification of collagen-rich fibrosis using hematoxylin–eosin staining and mucosal cholesteatosis. Very focal active inflammatory infiltrates are present; (**B**) Warthin–Starry silver staining of the section shown in panel A inside the white square; (**C**) Immunohistochemistry using a monoclonal antibody against *Helicobacter pylori* showing red, partly intracellular positive signals. In the right panel, *H. pylori-*like spiral shaped bacteria of approximately 2–3 μm in length are visible (blue arrows). Coccoid bacteria with up to 1 μm in diameter are also present (red arrows).

To corroborate the above findings, the gallbladder tissue samples were subjected to microscopic investigation. First, the specimens were stained with hematoxylin–eosin and examined independently by two attending pathologists, who specialized in biliary diseases. Acute cholecystitis was diagnosed by the presence of predominantly mononuclear inflammatory infiltrates, fibrosis with thickening of the gallbladder wall, cholesteatosis, and metaplastic changes. Lymphoid aggregates were found in the wall of the gallbladder but not within the mucosa. Remarkably, the bacteria that were observed in the tissue were primarily bended, curved, and spiral-shaped bacteria that were in close association to the epithelial cells (Figure 2A, blue arrows). Suspected Gram-negative *H. pylori* were approximately 0.5–1 µm in diameter and varied in length from 2–3 µm. In addition, spherical round-shaped bacteria were observed, which could either represent Gram-positive species or coccoid forms of *H. pylori* (red arrows). These morphological results suggested the presence of live, spiral-shaped, and spherical bacteria in the gallbladder tissue, in agreement with the culture results and their above described identification. Notably, these bacteria were mostly detected focally and only in certain locations.

The gallbladder tissue was further subjected to Warthin–Starry silver staining, which revealed the presence of bended, curved, and spiral-shaped bacteria, as well as some spherical bacterial cells in the close vicinity of epithelial cells in the mucosa (Figure 2B). These observations were very similar to the results from hematoxylin–eosin staining and confirmed the presence of different types of bacteria. Finally, to identify and localize putative *H. pylori* in the tissue, samples were subjected to immunohistochemistry staining using anti-*H. pylori* antibodies. A positive red signal detected bended, spiral-shaped or coccoid bacteria, as expected (Figure 2C, blue and red arrows). These bacteria were mainly located on the epithelial cell surface and within the mucosal glands, scattered or aggregated.

### 2.4. Biochemical Characterization of Gallbladder H. pylori

**Figure 3:**
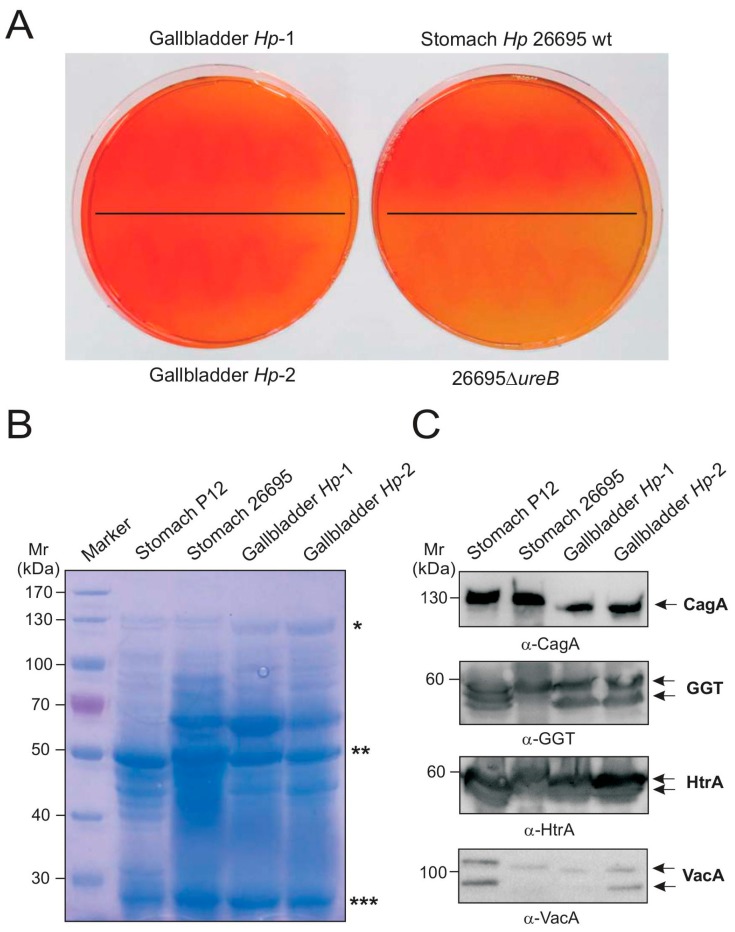
Urease test and Western blotting analysis of *H. pylori*-specific pathogenicity factors from gallbladder strains. (**A**) Two *H. pylori* isolates (Gallbladder *Hp-1* and *Hp-2*) were grown on acidified agar supplemented with urea (left samples). The observed color change from orange to red indicated that bacterial colonies were producing functional urease. The right samples represent positive controls. The color change occurred with the wild-type (wt) strain 26695 as expected, and was not observed with the negative control of an isogenic Δ*ureB* deletion mutant, indicating that functional urease enzyme was not being produced; (**B**) Protein profiling using Coomassie staining. Asterisks label the following protein bands: CagA (*), Urease B (**), and Urease A (***); (**C**) Western blots of two reference strains (P12 and 26695) and the two gallbladder isolates that identifies presence of *H. pylori* proteins CagA, VacA, GGT and HtrA.

The observation of *H. pylori* was surprising as there are no reports on the presence of these bacteria in gallbladders from patients from the Western hemisphere. To exclude artifacts, we screened for *H. pylori* urease, a telltale enzyme which bacteria express at high levels to neutralize the gastric pH in the lumen [17] and controls the inflammasome in immune cells [18]. For this purpose, bacteria were grown on selective acidified agar plates supplemented with urea, the substrate of *H. pylori* urease [17]. These experiments showed that two gallbladder isolates (*Hp-1* and *Hp-2*) expressed functional urease enzymes, with activity indistinguishable from that of the fully sequenced and stomach-derived *H. pylori* control strains 26695 and P12 [19,20]. In contrast, retarded growth and no urea hydrolysation was observed in a Δ*urease* knockout mutant of the reference strain that was included as a negative control (Figure 3A), or in the strains putatively identified as *S. saprophyticus* and *C. urinapleomorphum* (our unpublished data). To further characterize these gallbladder *H. pylori* isolates, we performed protein profiling of total cell lysates using Coomassie staining. Bands migrating at positions typical of highly expressed proteins CagA, Urease A, and Urease B [21] were identical between *Hp-1*, *Hp-2*, and the two *H. pylori* control strains (Figure 3B). Furthermore, Western blotting experiments using specific antibodies [22,23] confirmed the presence of several other well-known *H. pylori*-specific pathogenicity factors, including the typical gamma-glutamyl transpeptidase GGT, serine protease HtrA, the vacuolating cytotoxin VacA, as well as CagA proteins (Figure 3C, arrows). Thus, various independent methods clearly confirmed the successful isolation of live *H. pylori* from the gallbladder.

### 2.5. Genetic Comparison of Stomach and Gallbladder H. pylori

**Figure 4:**
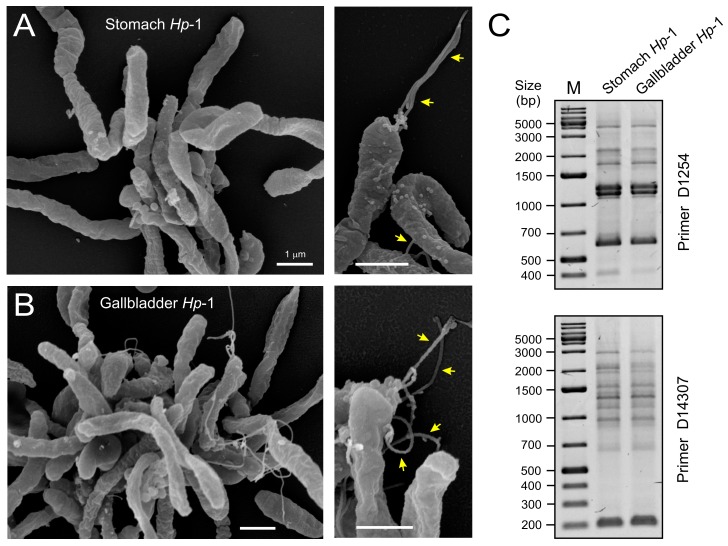
Scanning electron microscopic and genetic analyses of the stomach and gallbladder *H. pylori* isolates. High resolution scanning electron microscopy of the cultures obtained from the stomach (**A**) and gallbladder (**B**) of the same patient revealed spiral-shaped *H. pylori* bacteria. Arrows in the enlarged sections indicate typical monopolar flagella being present; (**C**) PCR-based randomly amplified polymorphic DNA (RAPD) produced identical fingerprints for the two *H. pylori* strains isolated from stomach and gallbladder. This method uses a set of single indicated primers (D1254 or D14307, top and bottom), which arbitrarily anneal and amplify genomic DNA resulting in strain-specific fingerprinting patterns. M = DNA size marker.

Lastly, we compared the *H. pylori* isolates of the stomach and gallbladder from the same patient phenotypically and genetically. Isolates subjected to field-emission scanning electron microscopy [24] revealed spiral-shaped *H. pylori* organisms with high similarity between the gastric (Figure 4A) and gallbladder samples (Figure 4B). These candidate *H. pylori* were approximately 0.5–0.8 µm in diameter, varied in length from 2–3 µm, and had typical monopolar flagella (yellow arrows). The genetic profiles of these strains were analyzed using random amplified polymorphism DNA (RAPD) fingerprinting method as described in ref. [25,26]. Because clinical *H. pylori* typically display DNA sequence diversity between individuals, different isolates are easily distinguishable by RAPD, even with a single RAPD primer [27]. This analysis produced identical fingerprinting patterns for all isolates (Figure 4C). To further confirm these findings, we amplified a 1.6 kb fragment of the 3′end in the *cagA* gene containing the EPIYA phosphorylation sequences and subjected the PCR products to sequencing. The results also showed identical *cagA* sequences for every strain and revealed a typical Western-type EPIYA-motif ABC arrangement (our unpublished data). Together, these data suggest that a single *H. pylori* strain of Western origin colonized both the stomach and the gallbladder.

## 3. Discussion

Infections of the human gastrointestinal tract have been proposed to frequently propagate to the gallbladder [3,28,29]. Recurrent microbial infections may play a role in the development of gallstones and contribute to inflammation and acute cholecystitis in patients. For example, acute calculous cholecystitis caused by an impacted gallstone is often complicated by a secondary bacterial infection, representing a major cause of morbidity and even mortality [30]. In addition, a wide variety of pathogenic microbes can be associated with acute acalculous cholecystitis, a less common, but potentially more severe form of acute cholecystitis [1,3,4,5,7]. As in the present case, calculus and gallbladder diseases are regularly treated by cholecystectomy [28]. Our report represents the first description of a combination of live *S. saprophyticus*, *C. urinapleomorphum*, and *H. pylori* from the gallbladder of a patient with gallstones and symptoms of cholecystitis.

*Staphylococcus saprophyticus*, a Gram-positive, coagulase-negative *Staphylococcus* species, is a regular cause of community-acquired urinary tract infections [31,32]. *S. saprophyticus* is commonly isolated from food such as meat, cheese, and vegetables, and often colonizes the human and animal gastrointestinal and genital tracts. *S. saprophyticus* causes about 10 to 20% of urinary tract infections, and patients commonly exhibit symptomatic cystitis. Similarly, *Corynebacterium urinapleomorphum* strain Marseille-P2799^T^ (CSURP2799) has been isolated from a urine sample of a two-months-old child with gastroenteritis [33], while *H. pylori* is commonly found in the human stomach and is responsible for diseases ranging from gastritis to severe malignancies [34]. Attempts to identify other natural reservoirs or routes by which *H. pylori* is transmitted to the stomach have been widely unsuccessful [35,36]. Some studies from Asia have reported the presence of *H. pylori* in the gallbladder of cholecystitis patients [2,5,6,7,8]. However, to date, it was not clear if this remarkable colonization route occurs in patients from Western countries, too.

Here, we unequivocally identified *H. pylori* in the gallbladder of a German patient. To our knowledge, this is the first report on the discovery of viable *H. pylori* from a gallbladder sample of a patient from the Western hemisphere, a phenomenon which deserves further investigation with large patient cohorts. Some of the bacteria were seen as intracellular, which is in agreement with earlier studies showing invasive *H. pylori* in gastric epithelial cells [37]. Using RAPD fingerprinting, we could also confirm that the obtained gallbladder isolate was genetically indistinguishable from the one previously cultured from the stomach of the same patient. In addition, the *cagA* gene may represent a good marker as genome sequencing projects have never revealed two identical *cagA* genes from different strains. Here, we could demonstrate that the *cagA* gene sequences in the patient are identical from gallbladder and stomach, encoding a typical Western-type CagA with classical ABC composition in the EPIYA-motif phosphorylation sites, and not the ABD-type present in East Asian isolates [38,39]. Extra gastric colonization could explain why very sensitive stool tests are positive for *H. pylori,* while the stomach was proven to harbor no *H. pylori* bacteria at all, for example, after eradication with antibiotics. In addition, it should be mentioned that *H. pylori* has never been found before together with live *S. saprophyticus* or *C. urinapleomorphum* at the same infection site*.* The regular habitat of these pathogens is the gastrointestinal or urogenital tract of humans, respectively. It can therefore be proposed that a gastrointestinal pathway, rather than transport through the bloodstream or other routes of infection, may be involved in the colonization of the gallbladder.

The possible contribution of *S. saprophyticus*, *C. urinapleomorphum*, and *H. pylori* to gallbladder pathology is still unclear. Further studies are therefore necessary to investigate if this environment may represent a reservoir for survival and growth that could serve as a potential source for bacterial transmission. In this case, it is remarkable that the antibiotic therapy eradicated *H. pylori* from the stomach, but not from the gallbladder. The reason for this treatment failure is not clear and deserves further investigation.

## 4. Materials and Methods

### 4.1. Ethics Statement, Biopsy Preparation, and Immunohistochemistry

All studies on human biopsy specimens were reviewed and approved through the FAU Ethics Bureau at Erlangen/Germany (license 344-16 BC to S.B., 29 November 2016). The patient gave his informed consent for inclusion before he participated in the study. Routine biopsy specimens were fixed in 4% neutral buffered formalin and paraffinized in an increasing series of alcohol and xylene. The paraffin blocks were cut into 4-micron thick slices and stained with hematoxylin and eosin. For detection of *H. pylori,* a Warthin–Starry Silver stain was performed. In addition, α-*H. pylori* antibodies (clone SP48 rabbit monoclonal, Ventana Medical Systems, Tucson, AZ, USA) were used to identify and localize *H. pylori* in the biopsy samples. Incubation for about 1 h at 4 °C was carried out for binding of the α-*H. pylori* primary antibody as described [40]. Antibody detection was performed using the *ultra-view* Universal Alkaline Phosphatase Red Detection Kit (Tucson, AZ, USA) according to the manufacturer’s protocol (Ventana Medical Systems, Tucson, AZ, USA).

### 4.2. Bacterial Isolation

Biopsy specimens were collected in sterile Falcon tubes, incubated with brain heart infusion (BHI) medium (2.5 mL per 0.5 g material), and shaken at 37 °C for 15 min at 5000× *g*. The mixture was then centrifuged for 5 min at 500× *g* to remove larger particles and cell debris. Bacteria were then cultured in different amounts (25, 50, 100 or 200 μL) on various culturing media including Mueller–Hinton agar plates, GC agar plates with 10% horse serum, *H. pylori* selective agar plates, and Columbia agar plates with 5% sheep blood. We used the gas generating systems Anaerogen and Campygen (Oxoid-Fisher Scientific, Wesel, Germany) for incubation of the agar plates in anaerobic jars or cultured the plates under aerobic conditions at 37 °C [41,42]. These plates were incubated for 2–7 days. All single colonies were selected and grown for further studies.

### 4.3. Bacterial Gram-Staining

The method was utilized in the initial phase of studies to examine the homogeneity, morphological features, and pureness of all grown bacteria. For this purpose, bacterial samples were analyzed by the conventional Gram-staining method using crystal violet, Gram’s iodine, acetone–ethanol (50:50 ratio), and 0.1% basic fuchsin solution as described [43].

### 4.4. Bacterial Urease Test

All isolated *H. pylori* strains were grown on GC agar plates under standard conditions as described above. To investigate for active urease enzyme activity, the bacteria were transferred to selective acidified GC agar plates complemented by the urease substrate urea and phenol red as a pH indicator according to a described protocol [17].

### 4.5. DNA Isolation and 16S rRNA Gene Analysis

Bacterial DNA was isolated from all colonies using Wizard^®^ Genomic DNA Purification Kit (Promega, Madison, WI, USA) and subjected to PCR for the amplification of a ~1 kb segment of the 16S rRNA gene using universal primers 27F (5′-AGA GTT TGA TCM TGG CTC AG-3′) and 926R (5′-CCG TCA ATT CCT TTR AGT TT-3′), as well as 16S-FW (5′-GAA GAG TTT GAT CAT GGC TCA G-3′) and 16S-Rev (5′-ACG ACA GCC ATG CAG CAC CT-3′), respectively [12,13]. The 16S rRNA gene sequences from the various strains were determined by standard sequencing at GATC Biotech (Konstanz, Germany). The sequences from isolates 1, 2, and 3 were sent to a Nucleotide BLAST search (available online: https://blast.ncbi.nlm.nih.gov/Blast.cgi), and similar sequences were determined [43]. The results were downloaded and aligned using CLUSTAL in MEGA7 (available online: http://www.megasoftware.net) [14]. Similarity coefficients among sequences were performed using the unweighted pair group method and arithmetic average (UPGMA) [15]. The evolutionary distances were computed using the Maximum Composite Likelihood method [16] and are in the units of the number of base substitutions per site.

### 4.6. Protein Profiling

Total protein profiling of the bacteria was done using SDS polyacrylamide gel electrophoresis (SDS-PAGE) according to an adapted protocol [44]. In brief, prepared bacterial cell pellets of the obtained gallbladder isolates and gastric control *H. pylori* strains 26695 and P12 were suspended in 1× SDS-PAGE buffer and boiled for 5 min [36,45]. The samples were subjected to 8% SDS-PAGE gels.

### 4.7. Western Blotting and Antibodies

Bacterial samples were resolved in 6% and 8% SDS-PAGE gels, followed by Western blotting using the semidry blotting method. Mouse monoclonal anti-CagA antibodies were purchased from Austral-Biological (San Ramon, CA, USA). Polyclonal rabbit α-VacA antiserum was kindly provided by Prof. Timothy Cover (Nashville, TN, USA). Additional polyclonal rabbit antisera against the *H. pylori* virulence proteins HtrA and GGT were raised against conserved peptide residues (HtrA amino acids 90–103: DKIKVTIPGSNKEY and GGT amino acids 175–188: RQAETLKEARERFL) derived from *H. pylori* strain 26695 [22,46]. These antibodies were generated, purified, and prepared using standard protocols of the manufacturer (Biogenes GmbH, Berlin, Germany). Polyvalent goat anti-rabbit or anti-mouse immunoglobulins coupled to horseradish peroxidase were utilized as secondary antibodies (DAKO, Glostrup, Denmark). The immunoblots were developed using the ECL Plus Western blot kit (GE Healthcare, Buckinghamshire, UK) [47,48].

### 4.8. Field Emission Scanning Electron Microscopy

Bacteria were fixed for 1 h on ice in cacodylate buffer (0.1 mM cacodylate, 0.09 mM sucrose, 10 μM MgCl_2_, 10 μM CaCl_2_ at pH 6.9) containing 2% glutaraldehyde and 5% formaldehyde [49,50]. The solution was passed through a 0.21 μm sterile filter (Sigma-Aldrich (St. Louis, MO, USA). After subsequent washing steps in cacodylate buffer and TE buffer (1 mM EDTA, 20 mM Tris at pH 6.9), the specimen were dehydrated for 15 min on ice in each step using serial dilutions of 10%, 30%, 50%, 70%, 90%, and 100% acetone, respectively. The samples were then further incubated at room temperature, followed by incubation in fresh 100% acetone. The samples were afterwards subjected to a critical point drying step using liquid CO_2_ (CPD030; Bal-Tec, Balzers, Liechtenstein). All samples were subsequently covered with 10 nm gold–palladium films using the sputter coating method (SCD500; Bal-Tec). Specimen examination was performed by the Zeiss–Merlin field emission scanning electron microscope operating with Everhart–Thornley and in-lens SE-detectors in a 25:75 ratio at 5 kV acceleration voltage (Oberkochen, Germany).

### 4.9. RAPD Fingerprinting

We applied the RAPD fingerprinting methodology to distinguish the identified *H. pylori* isolates [27]. The procedure utilizes arbitrary oligonucleotide sequences to prime DNA fragments across the entire genome. Twenty ng of genomic DNA from each strain were used as template in PCR reactions of a total volume of 50 μL including 20 pmol of each primer (D1254 or D14307, respectively) [27]. PCR buffer, one unit Taq-DNA polymerase (Qiagen, Hilden, Germany), 250 mM dNTPs and sterilized double distilled water were added. A Peqlab Primus 96 advanced^®^ thermal cycler was used for amplification reactions. The cycling program was four cycles of (94 °C, 5 min; 40 °C, 5 min; 72 °C, 5 min), thirty cycles of (94 °C, 1 min; 55 °C, 1 min; 72 °C, 2 min), and a final incubation at 72 °C for 10 min.

### 4.10. CagA Gene PCR and Sequencing

To analyze the origin of the strains, EPIYA-motifs in different CagA proteins, *cagA* gene subfragments from *H. pylori* strains were amplified by PCR using primers 48F (5′-AAA GGA TTG TCC CTA CAA GAA GC-3′) and 38R (5′-CTC GAG ATT TTT GGA AAC CAC CTT TTG-3′), followed by purification via NucleoSpin^®^ Gel and PCR Clean-up columns (Macherey-Nagel, Dueren, Germany). Sequences of *cagA* subfragments from the various strains were determined by GATC Biotech. The obtained sequences were analyzed by CLUSTAL Omega multiple sequence alignment analysis (available online: https://www.ebi.ac.uk/Tools/msa/clustalo/) and a BLASTX search to determine the EPIYA-motif patterns (available online: https://blast.ncbi.nlm.nih.gov/Blast.cgi).
